# ImageGP: An easy‐to‐use data visualization web server for scientific researchers

**DOI:** 10.1002/imt2.5

**Published:** 2022-02-21

**Authors:** Tong Chen, Yong‐Xin Liu, Luqi Huang

**Affiliations:** ^1^ State Key Laboratory Breeding Base of Dao‐di Herbs National Resource Center for Chinese Materia Medica, China Academy of Chinese Medical Sciences Beijing China; ^2^ State Key Laboratory of Plant Genomics, Institute of Genetics and Developmental Biology, Innovation Academy for Seed Design Chinese Academy of Sciences Beijing China; ^3^ CAS Center for Excellence in Biotic Interactions University of Chinese Academy of Sciences Beijing China; ^4^ CAS‐JIC Centre of Excellence for Plant and Microbial Science, Institute of Genetics and Developmental Biology Chinese Academy of Sciences Beijing China

**Keywords:** amplicon, bioinformatics, data visualization, metagenome, microbiome, web server

## Abstract

Data visualization plays a crucial role in illustrating results and sharing knowledge among researchers. Though many types of visualization tools are widely used, most of them require enough coding experience or are designed for specialized usages, or are not free. Here, we present ImageGP, a specialized visualization platform designed for biology and chemistry data illustration. ImageGP could generate generalized plots like lines, bars, scatters, boxes, sets, heatmaps, and histograms with the most common input content in a user‐friendly interface. Normally plotting using ImageGP only needs a few mouse clicks. For some plots, one only needs to just paste data and click submit to get the visualization results. Additionally, ImageGP supplies up to 26 parameters to meet customizable requirements. ImageGP also contains specialized plots like volcano plot, functional enrichment plot for most omics‐data analysis, and other four specialized functions for microbiome analysis. Since 2017, ImageGP has been running for nearly 5 years and serving 336,951 visits from all over the world. Together, ImageGP (http://www.ehbio.com/ImageGP/) is an effective and efficient tool for experimental researchers to comprehensively visualize and interpret data generated from wet‐lab and dry‐lab.

## Highlights

- Publication‐quality visualization results.
- Easy to use and customize.
- Reproducible results with scripts.

## Graphical abstract

Representative visualization results of ImageGP. ImageGP supports 16 types of images (including heatmap, volcano plot, enrichment bubble plot) and four types of online analysis with up to 26 parameters for customization.

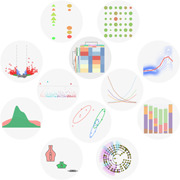

## INTRODUCTION

The advancements of various high‐throughput omics technology like metagenome, transcriptome, proteome, and metabolism generated an unprecedented amount of data. This leads to lots of challenges in data analysis and data explanation. Data visualization could enable researchers to explore, interpret, and present the results in a clearer and graceful way [1, 2, 3]. However, data visualization is not an easy job, especially for most wet‐lab scientists. The R programming language and related packages which could integrate data analysis and data visualization attract more and more usages [4]. Package ggplot2 [5] and other extension packages are the outstanding representatives. But for most researchers who have few programming skills, this type of operation is not easy. Specialized tools like Parallel‐Meta Suite integrates analysis and visualization together which supplies feasibility but lacks some customizability [6]. Other tools like Excel, GraphPad, Origin, and MATLAB could supply some convenience but charge a lot. Additionally, this software still needs lots of mouse clicking and interface transitions, giving hard efforts for reproducing the results.

Here we present the online web server ImageGP to try to smooth the data visualization process. ImageGP contains 16 subfunctions for data visualization or data analysis. Most of these plotting functions are based on the R programming language and popular packages, which could generate similar visual styles for publication just as if you are running the real R codes but without the real coding process. Besides, all the plotting codes of ImageGP are encapsulated in bash scripts and saved in Github (https://github.com/Tong-Chen/s-plot), which could be run in batch or for reproducible research in the local computer with either operating system like Windows/Mac/Linux. Also, most of these functions especially the four ones PICRUSt [7], LEFSe [8], FAPROTAX [9], BugBase [10] could use the output of EasyAmplicon or other popular amplicon pipelines as input, which further reduced the burden of data transformation. ImageGP bridges the data matrix and visualization graphs, which could be a great facility for scientific data presentation.

## METHODS

The ImageGP is implemented as a web application using Javascript, HTML, and bootstrap for front‐end development. High‐level web framework ThinkPHP is used for backend data preprocess and data analysis. Most plots are generated based on the R programming language with packages including ggplot2 [5], pheatmap, ggbeeswarm, VennDiagram [11], ggpubr, ggrepel, vegan [12], UpSetR [13], ggfortify [14], riverplot, and other assistant packages. All the codes are shared in https://github.com/Tong-Chen/s-plot. FAPROTAX function is based on the original FAPROTAX package. LEFSe function is based on the modified version of the original LEFSe in https://github.com/Tong-Chen/lefse. PICRUSt function is based on the first version of PICRUSt. BugBase is based on the original BugBase R script. Essential encapsulation using in‐house python scripts or bash scripts was coded for online running.

## RESULTS

### Overview of ImageGP

**Figure 1:**
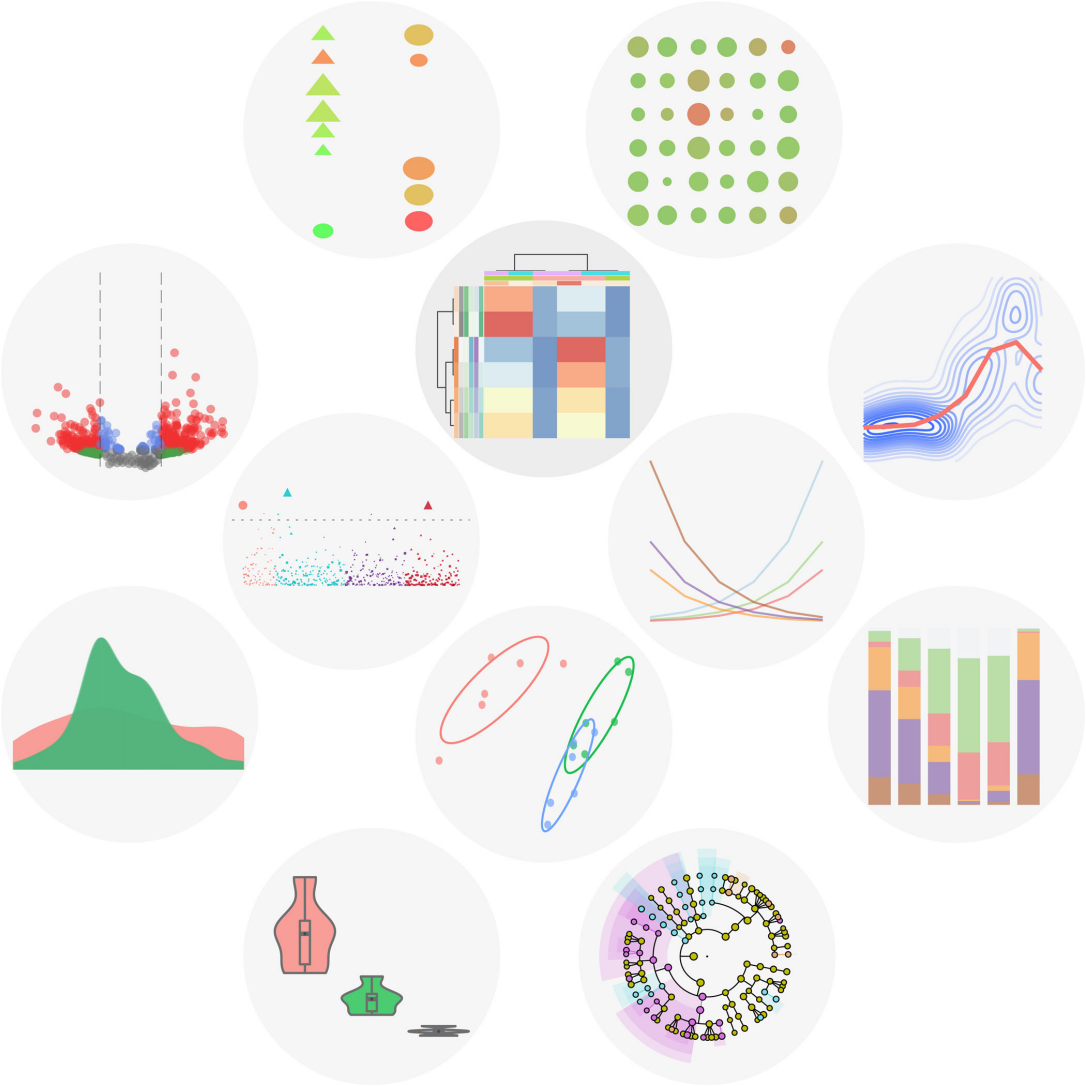
Representative visualization results of ImageGP

ImageGP provides 16 subfunctions for data visualization or data analysis (Figure 1). ImageGP is designed with a much concise interface with input text area at the beginning and only essential parameters are unfolded for user operation, which could reduce the pressure of understanding the meanings of all parameters. The input data could be just pasted from text‐editors or Excel tables and their format would be checked on blur (just after the data matrix is pasted). Common data errors like non‐numbers, irregular matrix, unsuitable column names, duplicate row names could be checked and got detailed error hints for instant modification before moving on to the next steps.

All essential parameters are designed as a drop‐down list for user selection instead of typing to avoid input errors and also to give some hints about what values should be given here. Parameters with value changes would be highlighted in the yellow background for distinguishing. For some specific data tables like a wide‐format matrix, no essential parameter is needed. Users just need to paste the data in and click submit to get the results.

Demo data, demo parameters, and demo results are processed into the slideshows as the most easy‐to‐read tutorials. At least one demo data is saved in the input text area for illustrating the needed data format or just could be used to test if the function is working. The demo button could also show the data format and parameter usages.

### Case studies and results

#### Case I: Heatmap of gene expression profiles

**Figure 2:**
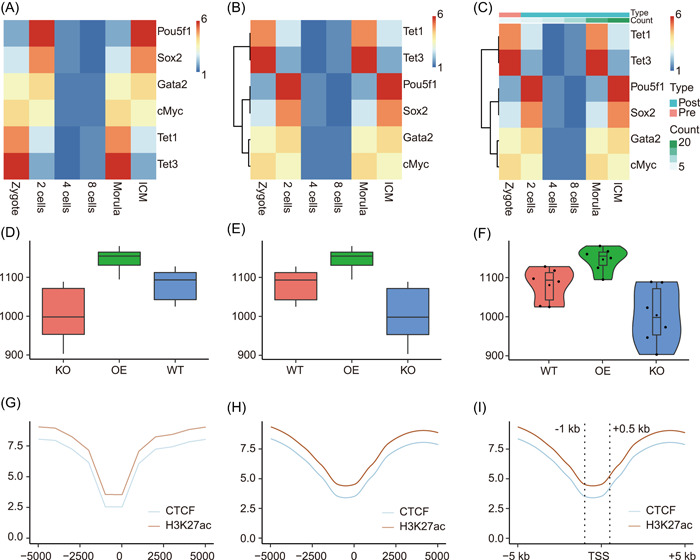
Examples of ImageGP output. (A–C) Visualization output of subfunction Pretty heatmap. Each row represents one gene and each column represents one sample. Color saturation represents gene expression abundances from low to high as indicated by the color bar from blue to red. (D–F) Visualization output of subfunction boxplot. Boxplot showing alpha diversity of three sample groups. Each point represents the alpha diversity index for each sample. (G–I) Visualization output of subfunction Line plot. Line plots showing the meta‐gene profile of CTCF (CCCTC binding factor) binding and H3K27ac in the flanking 5 kb of transcription start sites (TSS)

Heatmap may be the most popular visualization graph for showing matrix data such as differentially expressed genes, marker species, or metabolites abundances. In line with clustering, the underlying data patterns would be shown clearly. We use the demo data (expression matrix of six genes in six stages) as an example. For the simplest way, we just need to paste the data into the input text area and click “Submit” to get the first version heatmap (Figure 2A). Then we could perform row clustering to put genes with similar expression patterns together (Figure 2B). Next, we could add metadata information as columns annotations to show sample attributes (Figure 2C). More parameters could be tuned as demanded.

#### Case II: Boxplot for alpha diversity

Boxplot is the recommended visualization type for comparing data distribution. One application of boxplot in microbiome data analysis is to illustrate alpha diversity differences among multiple niches [15]. We use the alpha diversity matrix generated by the EasyAmplicon pipeline as an example. One additional operation is that we need to paste the metadata matrix with the alpha diversity matrix together in Excel. The concatenated data matrix could be just pasted into the boxplot subfunction of ImageGP. Two essential parameters should be selected from the drop‐down list. The *Legend variable* parameter is to set biological group information of samples (here we have three groups, *WT*, *KO*, and *OE*). The *Y‐axis variable* parameter is set to which alpha diversity index to be illustrated. Then we just need to click the *PLOT* button and would get Figure 2D. Normally we would like to put the wild‐type group (WT) as the first one and this could be achieved by selecting group order in the *Legend variable order* parameter to get Figure 2E. Violin plot and jitter plot showing sample points could also be generated by setting the *Plot type* parameter (Figure 2F).

#### Case III: Meta‐gene profiles

When focusing on transcription factor binding or histone modification patterns, the meta‐gene profile along flanking regions of transcription starting sites would be plotted [16]. The input data could be as simple as only two columns. The first column is the bins, and the second column contains the binding strength in each bin. Here we added a third column containing the names of binding proteins or histone modification to plot multiple profiles together as shown in the *Demo* data. This is a standard long format matrix. Then we set the *X‐axis type* as “Continuous variable,” *X‐axis variable* as “Pos” in the dropdown selection, *Legend variable* as “variable” as for demo data. After clicking the *PLOT* button, the resulting picture would be shown below to check the visualization effects (Figure 2G). Next, we specialize some additional parameters to smooth lines, add vertical lines, and give meaningful *x*‐axis labels to get a publication‐ready result (Figure 2H,I).

## DISCUSSION

ImageGP is not a new redevelopment of data visualization but a web server with much experience in scientific data illustration. It helped many wet‐lab researchers visualize data or analyze data in a much easier and efficient way. The R code used for plotting is open‐sourced and could be used as a getting started tutorial for R learners.

Next, we would continue maintaining and optimizing the function of ImageGP. Additionally, we are developing an upgraded platform that makes the deployment of subfunctions more easily to promote more tools to be added. All R codes would be reorganized to form an R package for more spreadable usages and more functions would be added.

## CONFLICT OF INTERESTS

The authors declare no conflict of interests.

## AUTHOR CONTRIBUTIONS

Tong Chen developed the scripts, designed the web server, and wrote the manuscript, and the other authors have tested the web server, suggested amendments, and revised the manuscript. Yong‐Xin Liu and Luqi Huang supervised this project and revised the manuscript. All authors have read the final manuscript and approved it for publication.

## Data Availability

The demo data used in this paper can be viewed and downloaded from ImageGP (http://www.ehbio.com/ImageGP/). The scripts used are saved in GitHub https://github.com/Tong-Chen/s-plot or Gitee https://gitee.com/ct5869/s-plot.
